# Co-introduction of plasmids harbouring the carbapenemase genes, *bla*_NDM-1_ and *bla*_OXA-232_, increases fitness and virulence of bacterial host

**DOI:** 10.1186/s12929-019-0603-0

**Published:** 2020-01-03

**Authors:** Haejeong Lee, Juyoun Shin, Yeun-Jun Chung, Myungseo Park, Kyeong Jin Kang, Jin Yang Baek, Dongwoo Shin, Doo Ryeon Chung, Kyong Ran Peck, Jae-Hoon Song, Kwan Soo Ko

**Affiliations:** 1 Department of Molecular Cell Biology, Sungkyunkwan University School of Medicine, Suwon, 16419 Republic of Korea; 2 Department of Microbiology, College of Medicine, The Catholic University of Korea, Seoul, 06591 Republic of Korea; 3 Precision Medicine Research Center, Integrated Research Center for Genome Polymorphism, College of Medicine, The Catholic University of Korea, Seoul, 06591 Republic of Korea; 4 Department of Integrative Biotechnology, College of Biotechnology and Bioengineering, Sungkyunkwan University, Suwon, 16419 South Korea; 5 Department of Anatomy and Cell Biology, Sungkyunkwan University School of Medicine, Suwon, 16419 Republic of Korea; 6 Asia Pacific Foundation for Infectious Diseases (APFID), Seoul, 06351 Republic of Korea; 7 Division of Infectious Diseases, Samsung Medical Center, Sungkyunkwan University School of Medicine, Seoul, 06351 Republic of Korea

**Keywords:** Carbapenemase, NDM-1, OXA-232, Plasmid, Plasmid paradox

## Abstract

**Background:**

Bacterial isolates with multiple plasmids harbouring different carbapenemase genes have emerged and been identified repeatedly, despite a general notion that plasmids confer fitness cost in bacterial host. In this study, we investigated the effects of plasmids with carbapenemase genes on the fitness and virulence of bacteria.

**Methods:**

Different plasmids harbouring the carbapenemase genes, *bla*_NDM-1_ and *bla*_OXA-232_, were isolated from a carbapenem-resistant *K. pneumoniae* strain. Each plasmid was conjugated into the *Escherichia coli* strain DH5α, and a transconjugant with both plasmids was also obtained by transformation. Their in vitro competitive ability, biofilm formation, serum resistance, survival ability within macrophage and fruit fly, and fly killing ability were evaluated.

**Results:**

The transconjugants with a single plasmid showed identical phenotypes to the plasmid-free strain, except that they decreased fly survival after infection. However, significantly increased fitness, virulence and biofilm production were observed consistently for the transconjugant with both plasmids, harbouring *bla*_NDM-1_ and *bla*_OXA-232_.

**Conclusions:**

Our data indicate that bacteria carrying multiple plasmids encoding different carbapenemases may have increased fitness and virulence, emphasizing the need for diverse strategies to combat antimicrobial resistance.

## Introduction

Carbapenems are antibiotics used for the treatment of severe infections caused by multidrug resistant gram-negative pathogens [1]. However, carbapenem-resistant isolates have emerged as important causes of morbidity and mortality, among hospital-acquired and long-term care-associated infections [2]. Particularly, the carbapenemase-producing *K. pneumoniae* isolates have become a public health problem globally due to their transmission mechanism and the limited therapeutic options available [1, 3].

In addition to *K. pneumoniae* carbapenemase (KPC), the New Delhi metallo-β-lactamase (NDM) and class D oxacillinases (OXA)-48 group carbapenemases are becoming the main causes underlying carbapenem resistance in *K. pneumoniae* [4, 5]. Since the first report in 2009 [6], NDM-1 and its variants have been identified in various bacterial species worldwide [7]. OXA-48 carbapenemases were initially identified in Istanbul, Turkey in 2001 [8, 9]. OXA-232, a variant of OXA-48, was first reported in an *E. coli* and two *K. pneumoniae* isolates [10].

Recently, *K. pneumoniae* strains co-producing NDM-1 and OXA-232 have been reported in several countries [11–13]. In the strains, the *bla*_NDM-1_ and *bla*_OXA-232_ genes are in different plasmids [14]. Compared to the plasmid bearing *bla*_NDM-1_, the plasmid with *bla*_OXA-232_, a ColE-type plasmid, is very small (about 6000 bp) [14]. The associations between small and large plasmids are common across a wide range of bacterial phyla [15]. Hence, positive epistasis between co-infecting plasmids minimizes the cost associated with carrying multiple plasmids in bacterial populations [15].

In this study, we investigated the contributions of the single or dual presence of plasmids bearing *bla*_NDM-1_ and *bla*_OXA-232_ on the fitness and virulence in bacteria, using the plasmids from a *K. pneumoniae* strain isolated from the blood sample of a patient in a Korean hospital.

## Materials and methods

### Bacterial strains and plasmids

A *K. pneumoniae* strain, M5, co-producing NDM-1 and OXA-232 was obtained from the blood sample of a patient, a 53-year-old man, who underwent liver transplantation for hepatocellular carcinoma, in Samsung Medical Centre (Seoul, South Korea). The strain M5 belongs to sequence type 14. Three plasmids with sizes of 253 kb (pKPM501), 250 kb (pM5_NDM), and 6 kb (pM5_OXA) were identified. The whole sequences were determined through next-generation sequencing method with PacBio RSII platform and de novo assembly was done with bioinformatics softwares (HGAP3, FALCON, and CANU) [16]. The plasmids containing *bla*_NDM-1_ (pM5_NDM) and *bla*_OXA-232_ (pM5_OXA) were transferred from the *K. pneumoniae* isolate M5 to the streptomycin-resistant (STR^R^) *E. coli* DH5α as a recipient. Conjugation mixtures were incubated overnight at 37 °C and plated on selective agar, resulting in two transconjugants, namely, DH5α::pM5_NDM and DH5α::pM5_OXA. Next, pM5_OXA was extracted from the transconjugant DH5α::pM5_OXA using a Qiagen Plasmid Mini kit (Qiagen, Hilden, Germany) and transformed into DH5α::pM5_NDM by electroporation [17], resulting in a transconjugant having two plasmids concurrently, DH5α::pM5_NDM + pM5_OXA. The three successful transconjugants, namely, DH5α::pM5_NDM, DH5α::pM5_OXA, and DH5α::pM5_NDM + pM5_OXA, were selected by plating onto MacConkey agar containing 0.25 mg/L of meropenem and 200 mg/L of streptomycin, and further confirmed by PCR with the primers for *bla*_NDM-1_ (forward, 5′-GGTTTGGCGATCTGGTTTTC-3′ and reverse, 5′-CGGAATGGCTCATCACGATC-3′) and *bla*_OXA-232_ (forward, 5′- GGCTGTGTTTTTGGTGGCAT-3′ and reverse, 5′-CGGTCAGCATGGCTTGTTTC-3′). In addition, we confirmed the absence of IncFIB plasmid with *bla*_CTX-M-15_ by PCR with the primers (forward, 5′-GCTGTCGCCCAATGCTTTAC-3′ and reverse, 5′-GGCGGACGTACAGCAAAAAC-3′).

### Antimicrobial susceptibility testing

The minimum inhibitory concentrations (MICs) of 12 antimicrobial agents including imipenem, meropenem, cefotaxime, ceftazidime, ampicillin, gentamicin, amikacin, ciprofloxacin, tetracycline, trimethoprim-sulfamethoxazole, piperacillin-tazobactam, and colistin were determined using a broth microdilution method following the Clinical and Laboratory Standards Institute (CLSI) guidelines [18]. *E. coli* ATCC 25922 and *Pseudomonas aeruginosa* ATCC 27853 were used as controls. All the tests were performed in duplicate, and each test included three biological replicates per strain.

### Competition assay

To assess the impact of the plasmids on bacterial fitness, we determined the relative fitness of the transconjugants against the *E. coli* strain DH5α. The competition assay was performed using a previously described method, with slight modifications [19]. The overnight cultures of the *E. coli* strain DH5α and one of the three transconjugants were inoculated to obtain a 0.5 McFarland standard and mixed at a 1:1 ratio in 10 mL of LB both, and incubated at 37 °C for 24 h with shaking. The number of cells for each strain was determined by spreading serial 10-fold dilutions onto LB agar plates with or without 0.25 mg/L imipenem. The competition index (CI) was defined as the ratio of carbapenem-nonsusceptible transconjugant colony forming units (CFUs) to the *E. coli* strain DH5α CFUs. Five independent competition experiments were performed.

### Biofilm formation assay

To measure the biofilm formation, 96-well microtiter plate assays were performed with crystal violet assay as described previously [20], with minor modifications. Briefly, the overnight bacterial cultures were diluted 1:100 in 10 mL of fresh LB medium and incubated until the bacterial suspension reached an OD_600_ of 0.5. Two hundred millilitres of the adjusted bacterial cultures were transferred to 96-well polystyrene microtiter plates and were incubated for 24 h at 37 °C. The cells were washed twice with phosphate-buffered saline (PBS) and stained with 1% crystal violet for 20 min at room temperature. The wells were dried and the bound dye was solubilized with 200 μL of 95% ethanol and quantified by measuring the absorbance at 600 nm. A well containing sterile LB without bacteria served as the negative control. Each experiment was performed in duplicate and repeated five times.

### Serum resistance assay

The serum resistance assays were performed as described previously [21]. Bacterial cultures were grown to mid-log phase (OD_600_ of 0.5). Then, 1-mL aliquots of the cultures were washed and resuspended with PBS. Then, 100 μL of the bacterial suspensions were added and mixed with 900 μL of PBS containing 20% normal human serum (NHS, Innovative Research, MI, USA), and the mixtures were incubated at 37 °C for 3 h with shaking. The number of surviving bacteria was determined by plating serial dilutions on agar plates and incubating at 37 °C overnight. Heat-inactivated human serum (HIS) was used as a control for determining the bactericidal effect of NHS. The survival rate was calculated as the ratio of the CFUs in the NHS to the CFUs in a bacterial suspension with HIS. All the experiments were performed five times and the results are expressed as survival percentage.

### Survival inside macrophages

Intra-macrophage survival assays were conducted with the macrophage-like cell line J774 A.1 as described elsewhere [22] with slight modifications. Macrophage cells were grown in Dulbecco’s modified Eagle’s medium (DMEM, Welgene) supplemented with 10% fetal bovine serum (FBS, Gibco) and 1% antibiotic-antimycotic solution (Thermo). A monolayer of 1 × 10^6^ J774A.1 cells was prepared in a 24-well tissue culture plate. After the cells were washed with Dulbecco’s phosphate buffered saline (DPBS, Welgene) and incubated in DMEM with FBS for 1 h, the overnight incubated bacterial cells were added at a ratio of 20 bacteria per macrophage (MOI 20). The cells were incubated for 30 min at 37 °C to permit phagocytosis and the free bacteria were removed by three washes with DPBS. Then, the cells were incubated for 1 h in the pre-warmed medium supplemented with 150 μg of gentamicin/mL to kill extracellular bacteria, and the wells were washed and incubated in the medium with 15 μg of gentamicin/mL. For the 0-h timepoint sample, the wells were washed and treated immediately by aspirating the medium and adding 500 μL of 1% Triton X-100 and 500 μL of DPBS. For the 4-h and 20-h time point samples, Triton X-100 was added at the desired time points. The content of each well was then diluted in DPBS and appropriate dilutions were plated on LB agar containing appropriate antibiotics. The percentage survival was obtained by dividing the number of bacteria recovered after 4 h and 20 h, by the number of bacteria present at time 0 and multiplying by 100. All the experiments were performed in duplicate.

### *Drosophila melanogaster* (fruit fly) infection

Fly infection was performed by the thoracic needle pricking method as described [21, 23], with minor modifications. Briefly, *D. melanogaster* Canton Special was cultured on standard cornmeal agar medium at 26 °C. Fifteen female flies 3 to 5 days old were infected with bacterial cultures at OD_600_ = 0.5 with ultra-fine needle (BD Bioscience). A pure PBS injection was used as a negative control and the fly mortality was monitored for up to 72 h post-infection. For quantification of viable bacteria, the infected flies were collected at 48 h post-infection, anesthetized with CO_2_, and three flies per bacterial isolate were individually ground in 100 μL of PBS with a Teflon pestle. Each resulting homogenate was serially diluted and plated onto LB agar containing appropriate antibiotics. The plates were incubated at 37 °C for 24 h and the number of CFU per fly was counted. Each experiment was performed four times.

### Statistical analyses

Statistical analyses were performed using Prism version 3.00 for Windows (GraphPad Software, San Diego, CA). The differences were assessed using the Student’s t-test, the one-way ANOVA with Tukey multiple comparisons test, and nonparametric Kruskal-Wallis test followed by Dunnett’s multiple comparison test. *P* value of less than 0.05 was considered statistically significant (*, *P* < 0.05; **, *P* < 0.001; ***, *P* < 0.0001).

### Accession numbers

The annotated sequences of pKPM501, pM5_NDM, and pM5_OXA have been submitted to the GenBank nucleotide sequence database (GenBank accession numbers CP031735, CP031736, and CP031737, respectively).

## Results

### Genetic characteristics of plasmids

The whole genome of strain M5 was sequenced using the PacBio RSII system, which identified 5,374,875 bp in the chromosome and three plasmids (pKPM501, IncFIB, 253,531 bp; pM5_NDM, IncHI/B, 250,351 bp; pM5_OXA, ColKP3, 6141 bp). The *bla*_NDM-1_ and *bla*_OXA-232_ carbapenemase genes were in two different plasmids, which were named as pM5_NDM and pM5_OXA. The G + C content of pM5_NDM was 46.4% and pM5_OXA was 52.2%. Their complete sequences were 250,351 bp and 6141 bp in length, with 283 and 7 coding genes, respectively (Table 1). Another plasmid, pKPM501, was 253,531 bp in length and had a G + C content of 51.2%, with 269 coding genes. While pM5_NDM bearing the *bla*_NDM-1_ gene also harboured additional antimicrobial resistance genes listed in Table 1, no other antimicrobial resistance gene except for the *bla*_OXA-232_ was identified in the smallest plasmid, pM5_OXA. *bla*_CTX-M-15_, an extended-spectrum β-lactamase gene, was identified in pKPM501.

**Table 1:** The genetic features of the chromosome and three plasmids in the *K. pneumoniae* strain M5

	Chromosome	pKPM501	pM5_ㅡ_NDM	pM5_ㅡ_OXA
Size (bp)	5,374,875	253,531	250,351	6141
GC %	57.4	51.2	46.4	52.2
Plasmid Inc. group	–	IncFIB	IncHI/B	ColKP3
Coding sequences	4905	269	283	7
Antimicrobial resistance genes	*bla*_OXA-1_ *bla*_SHV-28_ *aac(6′)-Ib-cr* *oqxB, oqxA* *fosA* *catB4* *dfrA1*	*bla*_TEM-1_ *bla*_CTX-M-15_ *aac(6′)-Ib* *aac(6′)-Ib-cr* *catA1* *dfrA14*	*bla*_NDM-1_ *bla*_OXA-1_ *aph(3′)-*Via *armA, aadA2* *aac(6′)Ib-cr* *catB4* *dfrA12* *mph(E), msr(E)* *sul1*	*bla*_OXA-232_

### Antimicrobial resistance

MICs of the transconjugants, a recipient, and their host strain were evaluated. The donor *K. pneumoniae* strain M5 co-producing NDM-1 and OXA-232 was not susceptible to most antibiotics except colistin (Table 2). As expected, the introduction of *bla*_NDM-1_ and *bla*_OXA-232_ genes in *E. coli* DH5α conferred decreased susceptibility to carbapenems (imipenem and meropenem) (Table 2). However, the carbapenem MICs in the transconjugants did not reach to the level of the donor. Particularly, DH5α::pM5_OXA showed imipenem and meropenem MICs of 1 mg/L and 0.25 mg/L, respectively. Moreover, the additional introduction of pM5_OXA into DH5α::pM5_NDM, resulting in a transconjugant with dual plasmids, did not increase the carbapenem MICs. Specifically, the imipenem MIC of DH5α::pNDM1 + pOXA232 was identical to that of DH5α::pNDM1 and the meropenem MIC increased two-fold, from 0.5 mg/L to 1 mg/L, which corresponds to susceptible category.

**Table 2:** Antimicrobial susceptibility profiles of the *K. pneumoniae* and *E. coli* strains used in this study

Strain	MIC (mg/L) (antimicrobial susceptibility category)^a, b^											
	IMI	MRP	CTX	CAZ	AMP	GEN	AMK	CIP	TET	SXT	P/T	CL
Donor (*K. pneumoniae* M5)	> 64 (R)	> 64 (R)	> 128 (R)	> 64 (R)	> 64 (R)	> 64 (R)	> 128 (R)	> 64 (R)	8 (I)	> 32/608 (R)	> 256/4 (R)	0.5 (S)
Recipient (*E. coli* DH5α)	0.125 (S)	0.06 (S)	0.125 (S)	0.5 (S)	4 (S)	1 (S)	4 (S)	0.06 (S)	0.5 (S)	0.25/4.75 (S)	4/4 (S)	0.06 (S)
DH5α::pM5_NDM	4 (R)	0.5 (S)	> 128 (R)	> 64 (R)	> 64 (R)	> 64 (R)	> 128 (R)	0.06 (S)	0.5 (S)	> 32/608 (R)	> 256/4 (R)	0.125 (S)
DH5α::pM5_OXA	1 (S)	0.25 (S)	4 (S)	0.5 (S)	> 64 (R)	4 (S)	16 (S)	0.06 (S)	0.5 (S)	2/38 (S)	> 256/4 (R)	0.06 (S)
DH5α::pM5_NDM +pM5_OXA	4 (R)	1 (S)	> 128 (R)	> 64 (R)	> 64 (R)	> 64 (R)	> 128 (R)	0.06 (S)	0.5 (S)	> 32/608 (R)	> 256/4 (R)	0.06 (S)

^a^*IMI* imipenem, *MRP* meropenem, *CTX* cefotaxime, *CAZ* ceftazidime, *AMP* ampicillin, *GEN* gentamicin, *AMK* amikacin, *CIP* ciprofloxacin, *TET* tetracycline, *SXT* trimethoprim-sulfamethoxazole, *P/T* piperacillin-tazobactam, *CL* colistin

^b^*R* resistant, *I* intermediate-resistant, *S* susceptible

DH5α::pM5_NDM and DH5α::pM5_NDM + pM5_OXA were also resistant to cefotaxime, ceftazidime, ampicillin, gentamicin, amikacin, trimethoprim-sulfamethoxazole, and piperacillin-tazobactam, but susceptible to the other antibiotics including ciprofloxacin, tetracycline, and colistin. DH5α::pM5_OXA was susceptible to most antibiotics except ampicillin and piperacillin-tazobactam.

### In vitro fitness

**Fig. 1:**
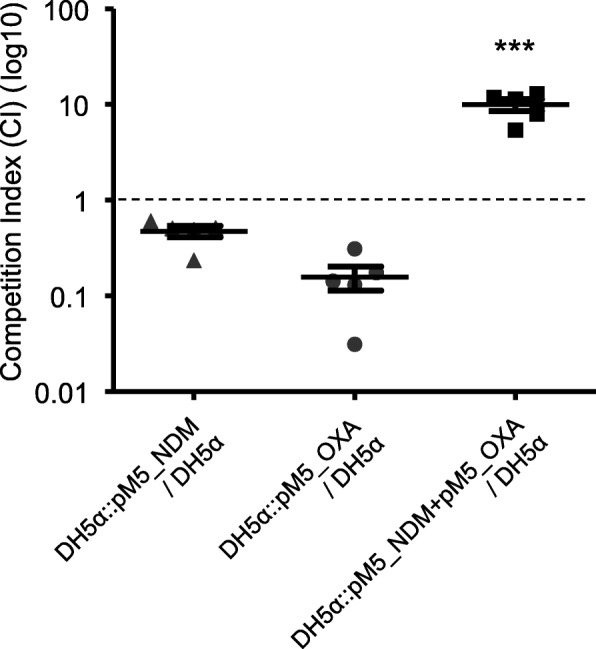
Fitness cost. Competitive fitness of the three transconjugants. A CI value less than 1 indicates a fitness defect, and a value greater than 1 indicates a fitness benefit. While the two transconjugants with a single plasmid (DH5α::pM5_NDM and DH5α::pM5_OXA) showed a fitness defect, a transconjugant with both the plasmids (DH5α::pM5_NDM + pM5_OXA) exhibited a marked fitness advantage. The differences were assessed using the two tailed Student’s t-test. ***, *P* < 0.0001

To investigate whether the propagation of plasmids harbouring *bla*_NDM-1_ and *bla*_OXA-232_ exhibit a fitness defect compared to the plasmid-free recipient strain, in vitro competition experiments were performed. In the media without antibiotics, the three plasmid-carrying transconjugants competed with the plasmid-free *E. coli* strain DH5α (Fig. 1). The transconjugants with single plasmid (DH5α::pM5_NDM and DH5α::pM5_OXA) showed CI values of less than 1. The transconjugant with both the plasmids, DH5α::pM5_NDM + pM5_OXA, outcompeted the plasmid-free strain DH5α (*P* < 0.0001) when analyzed with two tailed Student’s t-test, showing a mean CI value of 9.95.

### Biofilm formation and serum resistance

**Fig. 2:**
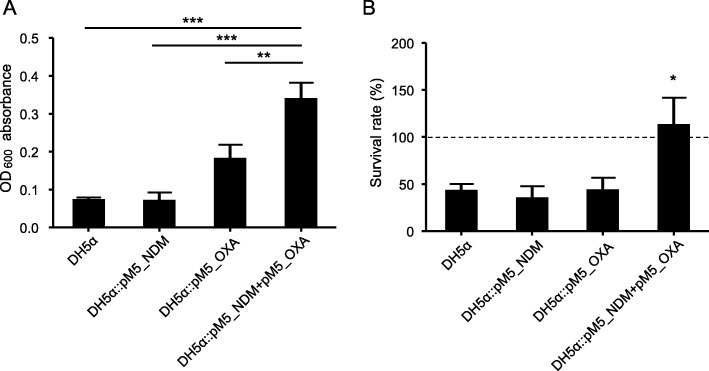
Results of biofilm formation and serum resistance. **a** Analysis of biofilm formation by DH5α and the three transconjugants (DH5α::pM5_NDM, DH5α::pM5_OXA, and DH5α::pM5_NDM + pM5_OXA). Biofilm was stained with crystal violet and quantified by measuring the absorbance at 600 nm. **b** Number of surviving bacterial colonies from human serum and those from heat-inactivated human serum as a control. We compared the biofilm formation activity and serum resistance statistically using one-way ANOVA with Tukey multiple comparisons test. *, *P* < 0.05; **, *P* < 0.001; ***, *P* < 0.0001

We performed biofilm formation assays on the recipient strain and three transconjugants (Fig. 2a). The transconjugant with pM5_NDM (DH5α::pM5_NDM) or pM5_OXA (DH5α::pM5_OXA) showed no difference in biofilm formation from the plasmid-free recipient strain DH5α. However, the transconjugant with both the plasmids formed significantly more biofilm compared to the plasmid-free recipient and two transconjugants containing single plasmid when analyzed with the one-way ANOVA with Tukey multiple comparisons test.

Survival rates of the recipient strain and the transconjugants were evaluated in the presence of NHS over a 3 h period (Fig. 2b). DH5α::pM5_NDM and DH5α::pM5_OXA showed no increase in the survival rates against human serum compared with the plasmid-free recipient strain DH5α. Only the transconjugant with both the plasmids, DH5α::pM5_NDM + pM5_OXA, exhibited a significantly higher survival rate against human serum than all the other strains (one-way ANOVA with Tukey multiple comparisons test, *P* < 0.05).

### Macrophage and fruit fly infection

**Fig. 3:**
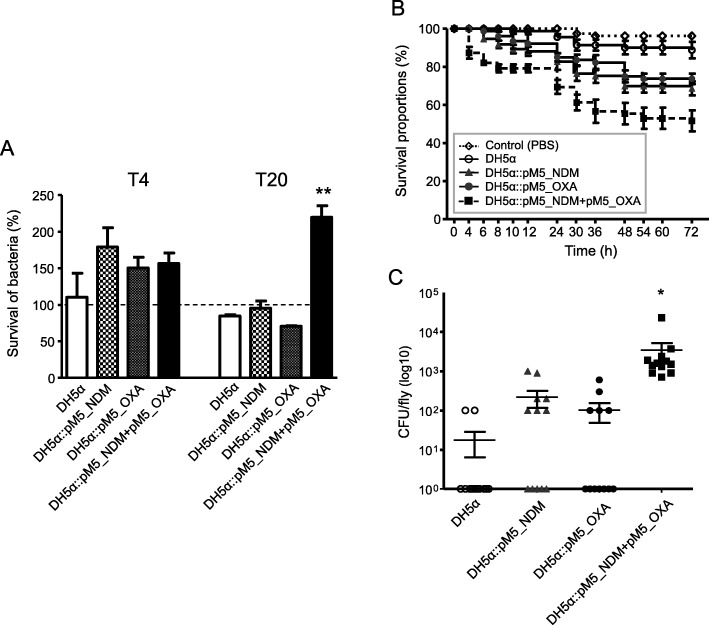
Results of macrophage and fruit fly (*D. melanogaster*) infections. **a** Survival rates of bacterial strains inside macrophage (J774A.1), which were measured at 4 h and 20 h of infection (T4 and T20, respectively). We compared the survival rates statistically using the one-way ANOVA with Tukey multiple comparisons test. **, *P* < 0.001 (**b**) Survival rates of flies infected with bacterial isolates at OD_600_ = 0.5. Fifteen flies were infected with each strain. **c** Number of surviving colonies of bacterial strains in the flies after 48 h of infection. Twelve fruit flies were used for each strain, and dots indicate the number of CFU in a single fly. The survival rates in the fly were analysed statistically using nonparametric Kruskal-Wallis test followed by Dunnett’s multiple comparison test (*P* < 0.05)

Survival of plasmid*-*carrying transconjugants inside macrophage was evaluated (Fig. 3a). In the intra-macrophage survival assay, the number of bacteria recovered after 4 h of infection (T4) did not differ among the four strains (one-way ANOVA, *P* > 0.05). However, after 20 h of infection (T20), DH5α::pM5_NDM and DH5α::pM5_OXA, as well as DH5α, did not multiply in macrophage, but the survival rate of DH5α::pM5_NDM + pM5_OXA was significantly higher (one-way ANOVA with Tukey multiple comparisons test, *P*, 0.0011).

We examined the survival rates of *D. melanogaster* against *E. coli* infections (Fig. 3b). The transconjugants with a single plasmid showed increased fly killing ability compared to the plasmid-free recipient strain DH5α and the transconjugant with both the plasmids (DH5α::pM5_NDM + pM5_OXA) showed a further increase in fly killing ability than those with a single plasmid. In addition, the number of viable bacteria isolates from flies after 48 h of infection was also measured (Fig. 3c). As in the experiment of fly killing, significantly more bacterial colonies survived in the flies infected with DH5α::pM5_NDM + pM5_OXA than the other strains (Kruskal-Wallis test followed by Dunnett’s multiple comparison test, *P* < 0.05)

## Discussion

It has been considered that plasmids generally impose a fitness cost on their bacterial hosts and thus, it is expected that they would not be retained in the cell in the absence of selective pressure. However, many studies have shown that plasmids can persist in bacterial populations over the long term, even in the absence of positive selection, which is referred to as the ‘plasmid paradox’ [24]. In this study, we showed that the simultaneous presence of two plasmids harbouring different carbapenemase genes increased the fitness and virulence of a bacterial host, although a single plasmid did not.

Each plasmid bearing a carbapenemase gene from a carbapenem-resistant *K. pneumoniae* strain, when introduced individually, did not increase the resistance level in *E. coli* to that in *K. pneumoniae*. The introduction of a plasmid harbouring *bla*_OXA-232_ did not impart carbapenem resistance comparable to that of the transconjugant with *bla*_NDM-1_. Previously, it was reported that the carbapenemase OXA-232 did not increase the MIC in *E. coli* transconjugants, as opposed to their effect in *K. pneumoniae* [10, 25]. Although the third plasmid, for example, a plasmid with *bla*_CTX-M-15_ in this study, may influence the fitness of the strain, this suggests that antibiotic resistance is determined by interactions between the resistance genes and bacterial host, and not by the existence of the resistance genes alone. In addition, it may also imply that the plasmid with carbapenemase gene can spread undetected, imparting resistance only under specific circumstances, such as in certain bacterial species and upon permeability defects in certain isolates [25].

One of the most interesting results in this study is that the transconjugant with both the plasmids showed increased fitness and virulence traits. Although many studies have shown that a single plasmid may increase fitness or virulence of the bacterial host [26–31], the transconjugants with a single plasmid did not in most tests in our study with the exceptions of reducing fly survival. In a previous study, the introduction of only *bla*_NDM-1_ did not increase the virulence or cytotoxicity in *E. coli* and *K. pneumoniae* transconjugants [20]. However, the *E. coli* strain with both plasmids, harbouring *bla*_NDM-1_ and *bla*_OXA-232_ which were isolated from a carbapenem-resistant *K. pneumoniae* strain, exhibited higher in vitro competitive ability, biofilm formation, serum resistance, and survival ability within macrophage and fruit fly, compared to transconjugants with a single plasmid. In addition, the transconjugant with both plasmids showed higher ability to kill fruit fly than those with a single plasmid as well as the parental strain with no plasmid. The high fitness and virulence of the bacterial strain with dual plasmids were consistent in all the experiments.

San Millan et al. [15] have shown that co-infection of a large plasmid and a small plasmid invokes positive epistasis, minimizing the cost associated with carrying multiple plasmids. In our study, pM5_NDM is a relatively large plasmid (about 250 kb) and pM5_OXA is a small one (about 6 kb), indicating that the positive epistasis between the two plasmids might occur. Although it is unknown which plasmid was introduced into the *K. pneumoniae* strain first and which plasmid was conjugated into the same strain later, our results suggest that the co-introduction of two plasmids harbouring carbapenemase genes can cause synergistic effect on the survival and spread of bacterial hosts.

In this study, it was not clear why the introduction of dual plasmids harbouring the carbapenemase genes increased the fitness and virulence. Several studies have shown transcriptional changes in the chromosomal and plasmid genes, and metabolites due to the introduction of plasmids [30, 32, 33]. However, no general mechanism has been proposed, indicating that the fitness effects of plasmids may be due to complex interactions [34].

Strains carrying multiple plasmids are relatively common [15]. In nature, NDM-1 and OXA-232-co-producing isolates have been identified repeatedly [11, 12, 35, 36]. In addition to *bla*_NDM-1_ and *bla*_OXA-232_, many isolates with multiple plasmids harbouring other antibiotic resistance genes have been reported [37, 38]. In addition, specific genes in the plasmid for instance, adhesion factors for biofilm formation, may affect the traits of the strain with plasmid, but we did not reveal which genes of plasmids affect the fitness or virulence traits, which would be further study. Because the fitness impact caused by plasmids may vary widely with different plasmids [28], the synergistic effect from the existence of dual plasmids bearing carbapenemase genes on survival, fitness, and virulence could not be generalized. However, our data suggest the possibility that bacterial strains with higher fitness and virulence traits would emerge and disseminate, due to the additional introduction of plasmid harbouring antibiotic resistance genes.

## Conclusions

A transconjugant with both plasmids harbouring *bla*_NDM-1_ and *bla*_OXA-232_, which originated from a carbapenem-resistant *K. pneumoniae*, exhibited increased fitness and virulent traits in terms of in vitro competition index, biofilm formation, in vitro serum resistance, survival within macrophage, and killing effect of *D. melanogaster* and survival within it. These data indicate that carbapenemase-producing gram-negative pathogens may disseminate even in the absence of antibiotic pressure and may cause more severe infections, emphasizing the need for diverse strategies to combat antimicrobial resistance.

## Data Availability

All materials are available by the corresponding author.

## Abbreviations

CFU: colony forming unit
CI: competition index
CLSI: Clinical and Laboratory Standards Institute
DMEM: Dulbecco’s modified Eagle’s medium
FBS: fetal bovine serum
HIS: heat-inactivated human serum
KPC: *Klebsiella pneumoniae* carbapenemase
LB: Luria-Bertani
MIC: minimum inhibitory concentration
MOI: multiplicity of infection
NDM: New Delhi metallo-β-lactamase
NHS: norman human serum
OXA: oxacillinase
PBS: phosphate-buffered saline
